# Color changes of esthetic orthodontic ligatures evaluated by orthodontists and patients: a clinical study

**DOI:** 10.1590/2177-6709.21.5.053-057.oar

**Published:** 2016

**Authors:** Edilene Kawabata, Vera Lucia Dantas, Carlos Brito Kato, David Normando

**Affiliations:** 1 Specialist in Orthodontics, Associação Brasileira de Ortodontia, Belém, Pará, Brazil.; 2 Professor in Orthodontics, Associação Brasileira de Ortodontia, Belém, Pará, Brazil.; 3 Adjunct professor, Universidade Federal do Pará (UFPA), School of Dentistry, Belém, Pará, Brazil.

**Keywords:** Esthetic orthodontic ligatures, Esthetic elastomers, Pigmentation Orthodontic treatment

## Abstract

**Objective:**

To evaluate *in vivo* changes in the color of esthetic elastomeric ligatures from different manufacturers.

**Methods:**

Four widely used commercial brands of elastomeric ligatures were selected and used in 20 adult patients in a split-mouth design. The ligatures were evaluated by orthodontists and patients in a double-blind manner on the day the ligatures were placed (T_0_) and 30 days after intraoral exposure (T_1_) by means of a system of staining scores. Groups were compared by Friedman test with *p* < 0.05.

**Results:**

Orthodontists and patients reported similar staining scores (*p* > 0.05). Results showed that all brands underwent significant staining when exposed to the intraoral environment. Modular-crystal Morelli^TM^ (Sorocaba, SP, Brazil) showed the highest degree of staining with the median reaching the maximum value (3); while the other brands (3M Unitek^TM^, American Orthodontics^TM^ and GAC Dentsply^TM)^ showed the median equal to 1 (*p* < 0.001). A large individual variability in the degree of staining was also found for all brands.

**Conclusions:**

All four brands of esthetic ligatures showed significant staining, which appeared to be more pronounced for the Morelli^TM^ brand. Changes in color of the elastomeric ligatures were perceived similarly by patients and orthodontists. The industry needs to improve the color stability of esthetic ligatures.

## RESUMO

**Objetivo:**

avaliar, *in vivo,* as alterações na cor de elastômeros estéticos de diferentes fabricantes.

**Métodos:**

quatro marcas comerciais de elastômeros amplamente utilizados foram selecionadas e utilizadas em 20 pacientes adultos, por meio desse estudo com delineamento do tipo boca dividida. As ligaduras foram avaliadas por ortodontistas e pacientes, com método duplo-cego, no dia que as ligaduras foram instaladas (T_0_) e após 30 dias em exposição ao meio intrabucal (T_1_), atribuindo escores ao manchamento dessas. Os grupos foram comparados pelo teste de Friedman, com *p* < 0,05.

**Resultados:**

os ortodontistas e pacientes relataram escores semelhantes de manchamento (*p* > 0,05). Os resultados mostraram que todas as marcas analisadas apresentaram alterações significativas na coloração, quando foram expostas ao ambiente intrabucal. O modelo Modular-cristal (Morelli, Sorocaba/SP, Brasil) mostrou o maior grau de manchamento, com a mediana atingindo o valor máximo (3), enquanto nas outras marcas (*3M Unitek, American Orthodontics* e *GAC Dentsply*) a mediana foi igual a 1 (*p* < 0,001). Também se verificou uma grande variabilidade individual no grau de manchamento, para todas as marcas.

**Conclusões:**

Todas as quatro marcas de elastômeros estéticos mostraram manchamento significativo, que pareceu ser mais pronunciado para a marca Morelli. As alterações de cor nos elastômeros foram percebidas de forma semelhante pelos pacientes e pelos ortodontistas. Assim, a indústria precisa melhorar a estabilidade de cor dos elastômeros estéticos.

## INTRODUCTION

Due to good color stability and improved adhesion, esthetic brackets have become very popular in Orthodontics in recent decades. Moreover, the demand of adult patients for orthodontic treatment performed with esthetic orthodontic brackets has increased substantially. Furthermore, as regards elastomeric ligatures used to tie the bracket/wire combination, clinical orthodontists are concerned and would like to make sure the ligatures' characteristics remain unchanged. Color changes caused by staining resulting from food ingestion or contact with intraoral fluids are particularly undesirable. These changes are due to swelling and discoloration when elastomers are exposed to the intraoral environment, and it is caused by buccal fluids and bacteria that fill up the spaces in the rubber matrix.^1^ ^,^ ^2^ ^,^ ^3^ In order to minimize the influence of some types of food affecting the color of elastomeric ligatures, metallic pigments have been added during the manufacturing process; however, they reduce the level of force released, impairing their elastomeric properties.^1^

Many *in vitro* studies have evaluated the effects of the intraoral environment on the elastomeric properties of elastomers, such as force decay, friction and dimensional changes.^1^ ^-^ ^7^ However, only a few *in vivo* studies have analyzed the behavior of orthodontic material after exposure to the intraoral environment,^8^ ^-^ ^11^ particularly the changes in esthetics of the elastomeric ligatures used.

*In vitro* studies have shown that esthetic elastomers become stained after being immersed in liquids with high susceptibility to pigmentation.^12^ ^-^ ^18^ However, these studies were conducted *in vitro*, which may not reflect the numerous factors present in the intraoral environment contributing to color change, such as the oral flora, temperature variation, the mechanical effect of brushing and solid and semi-solid food that cause pigmentation. Thus, clinical studies can yield a more realistic analysis of actual color changes taking place in orthodontic material after clinical use.^19^

Besides the need for clinical evaluation, patients' real perception of color changes undergone by elastomers is not yet known. Thus, this study aimed to investigate clinical changes in the color of esthetic elastomeric ligatures by means of direct visual analysis performed by both orthodontists and patients.

## MATERIAL AND METHODS

This study was approved by the Ethics Committee of the Institute of Health Sciences, under protocol #15958513.7.0000.0018. 

Four commercial brands of esthetic ligatures were selected based on a survey conducted on a social network of orthodontists. The following question was posted: "Which esthetic orthodontic ligature do you use in your practice?"

Relying on the collaboration of 94 orthodontists after ten days, the search resulted in the following brands: Obscure (3M Unitek^TM^, Monrovia, CA, USA) with 22 indications (30.13%); followed by American Orthodontics^TM^, pearl color (Sheboygan, Wisconsin, USA) with 21 indications (28.76%); Dentsply GAC^TM^, clear (New York, NY, USA) with 20 (27.39%) and Modular-crystal Morelli^TM^ (Sorocaba, SP, Brazil) with ten indications (13.69%).

**Figure 1:**
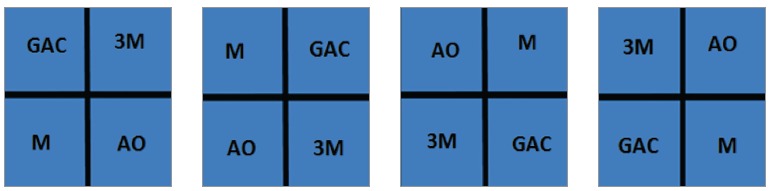
Random distribution by quadrant: M = Morelli^TM^, GAC = GAC^TM^, AO = American Orthodontics^TM^, 3M = 3M Unitek^TM^.

A split-mouth, double-blind, prospective study was designed. A convenience sample included 20 adult volunteer patients (14 females and 6 males), aged between 20 and 57 years old (mean age of 38.5 years). All patients were treated with esthetic ceramic orthodontic appliances from six different orthodontic practices. In each patient, the four brands were randomly distributed by hemiarch and remained in the oral environment for 30 days (Fig 1). Randomization was performed by means of BioEstat 5.3 software (Mamirauá Institute, Belém, Pará, Brazil).

The scoring process was performed while patients were using the ligatures. The ligatures were scored on the same day they were placed (T_0_), and after 30 days of exposure in the intraoral environment (T_1_). Evaluation was carried out visually and under cold light, both by patients using a mirror (n = 20) and orthodontists (n = 6), by the same examiner in T_0_ and T_1_, under the same light conditions. No patient received any guidance regarding food restrictions in their diet. Analysis involved the use of scores according to the degree of staining,^19^ in which: 0 = nonpigmented ligatures; 1 = slightly pigmented; 2 = moderately pigmented; and 3 = heavily pigmented.

Groups were statistically compared by Friedman test and ANOVA at 95% confidence level by means of BioEstat 5.3 software (Mamirauá Institute, Belém, Pará, Brazil).

## RESULTS

**Figure 2:**
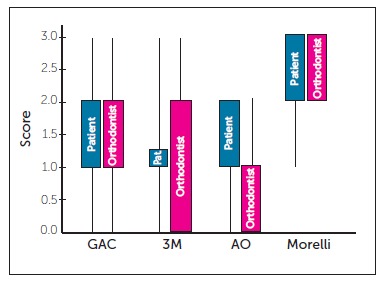
Box-plot of medians and IQR of the scores assigned to the four brands according to orthodontists' and patients' assessment (T_1_-T_0_).

Evaluation performed by orthodontists and patients was not statistically different. The median score for the four as-received elastomer was zero (*p* > 0.05). Patients and orthodontists evaluated similarly the staining of all brands at T_0_ (Table 1, Fig 2).

**Table 1:** Median, interquartile range (IQR) and *p*-value of four commercial brands evaluated at T_0_ by patients and orthodontists.

	GAC	3M	AO	Morelli	*p* value
	(n = 20)	(n = 20)	(n = 20)	(n = 20)	
Patients					
Median	0	0	0	0	0.96
IQR	0.25	1	0	0	
Orthodontist					
Median	0	0	0	0	0.14
IQR	0.25	1	1	0	

**Figure 3:**
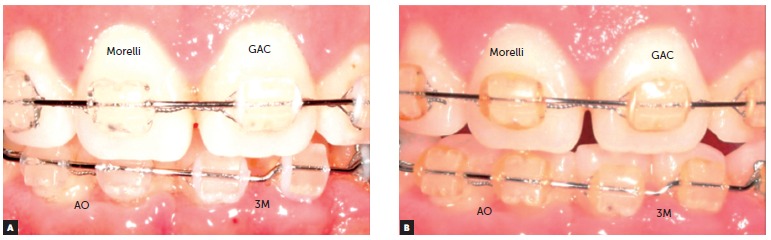
Change in color of the ligatures before (A) and after exposure to the intraoral environment for 30 days (B): note that whereas the ligatures manufactured by GAC^TM^, 3M^TM^ and American Orthodontics^TM^ show a pattern of less staining and are similar to each other, the ligature manufactured by Morelli^TM^ exhibits a more pronounced pigmentation.

After a 30-day period of intraoral exposure (T_1_), the four brands of elastomeric ligatures showed significant staining (Table 2). Differences were statistically significant in all groups (*p* < 0.001). The median observed at T_1_ was equal to 1.0 (slightly pigmented) for the three brands manufactured in the United States (GAC^TM^, 3M^TM^ and American Orthodontics^TM)^, while the Brazilian brand (Morelli^TM)^ had a score of 3.0 (heavily pigmented) assigned. The American products manufactured by GAC^TM^, 3M Unitek^TM^ and American Orthodontics^TM^ exhibited no significant differences when compared to one another (Table 2, Fig 2). However, a large variation in the degree of staining was observed in all brands. In patients' evaluation, GAC^TM^ and 3M^TM^ showed the greatest variation in staining, with scores ranging from 0 to 3, while American Orthodontics^TM^ had scores between 0 and 2 assigned. The Brazilian ligatures (Morelli^TM)^ received scores ranging from 1 to 3 (Fig 2). This variation observed by orthodontists was similarly reported by patients (Fig 2). 

**Table 2:** Median, interquartile range (IQR) and *p*-value of four commercial brands evaluated at T_1_-T_0_ by patients and orthodontists.

	GAC	3M	AO	Morelli	*p* value
	(n = 20)	(n = 20)	(n = 20)	(n = 20)	(Friedman)
Patient					
Median	1(a)	1(a)	1(a)	3(b)	< 0.0001
IQR	1	0.25	1	1	
Orthodontist					
Median	1(a)	1(a)	1(a)	3(b)	< 0.0001
IQR	1	2	1	1	
p value					
Ortho x patient	0.823	1	0.263	0.823	

Different letters indicate *p* < 0.05.

## DISCUSSION

Patients' concerns about facial esthetics and properly aligned teeth have been combined with increased life expectancy and quality of life to boost the demand for orthodontic treatment in adult patients; and with it, the demand for esthetic orthodontic appliances. Despite great improvement in the quality and stability of bracket color, esthetic appliances are faced with the challenge of changes that occur in the color of esthetic orthodontic ligatures when exposed to the intraoral environment. Thus, patients' complaints are frequent, given that the whole bracket/ligature combination becomes less esthetic, as elastomers undergo undesirable staining. 

Analyses of color changes in orthodontic ligatures are usually performed *in vitro*,^12^ ^-^ ^18^ which does not reflect reality. In this study design, clear elastomers are dipped into high-pigmentation fluids and analyzed after a given period of time. Unlike the clinical design used in the present study, laboratory studies are not affected by oral fluids, oral microflora, diet and oral hygiene. 

The *in vivo* model used in this study showed no significant difference between orthodontists and patients for all brands at the time the ligatures were placed (T_0_). The median was the same for all brands, i.e., all values were equal to zero; thus, the metal pigments added during the manufacturing process of some of the esthetic orthodontic ligatures had no significant impact on the assessment of patients and orthodontists alike. Thirty days after exposure to the intraoral environment, all ligatures exhibited some degree of staining, but no significant differences were found between GAC^TM^, 3M Unitek^TM^ and American Orthodontics^TM^. Morelli^TM^ ligatures obtained a median of 3, showing greater susceptibility to pigmentation. 

Moreover, it was observed that the pigmentation of elastomers made by the same manufacturer varied from patient to patient, which may have been related to diet and oral hygiene among the different experimental subjects. These results contradict the low pigmentation variability found in *in vitro* studies and further confirm the fact that individual factors can influence pigmentation intensity.

This clinical study differs from a previous *ex vivo* investigation^19^ whereby analysis of elastomer pigmentation was carried outside the intraoral environment. The ligatures were removed, photographed and then analyzed, which could lead to interference in the results, depending on the calibration when capturing the images. *In vivo* analyses of staining in esthetic orthodontic ligatures could provide more accurate results, since different brands are examined through a direct visual analysis in the intraoral cavity by orthodontists and especially by patients themselves. However, even when evaluating American Orthodontics^TM^ and Morelli^TM^ ligatures, results do not seem to differ.

Dental material science has focused on the properties of as-received material rather than on changes produced after intraoral exposure.^20^ The results of this study warrant the need for greater investment in research and technology to improve color stability of esthetic orthodontic ligatures. For now, a viable clinical option would be esthetic self-ligating brackets, which forestall the use of elastomeric ligatures. However, despite the elimination of undesirable staining in ligatures, metal clips, typically used in these attachments, contribute to substantial loss in appliance esthetics. Another option for the clinician, which happens to be more affordable than esthetic brackets, is the use of esthetic steel ligatures to tie the wire/bracket combination. The main drawback inherent to this method, however, is an increase in chair time compared to self-ligating brackets.^21^

## CONCLUSIONS

After examining four brands of esthetic elastomeric ligatures, all of them showed significant staining, which appeared to be more pronounced in Morelli^TM^ ligatures.^1^ Changes in color of elastomeric ligatures were perceived similarly by patients and orthodontists.
